# Urban road surface crack detection based on U-net and ResNeXt network

**DOI:** 10.1371/journal.pone.0347145

**Published:** 2026-04-21

**Authors:** Jun Qiao, Huabing Wang, Zidong Zhou, Yunwei Meng, Minghui Gong

**Affiliations:** 1 School of Mathematical Sciences, Beijing University of Posts and Telecommunications, Beijing, China; 2 New-type Rail Transit Design and Research Institute of China Railway Siyuan Survey and Design Group Co., Ltd., Suzhou, China; 3 China Railway Siyuan Survey and Design Group Co., Ltd., WuHan, China; 4 School of Traffic and Transportation Engineering, Changsha University of Science and Technology, Changsha, China; 5 Chongqing Key Laboratory of Intelligent Integrated and Multidimensional Transportation System, Chongqing Jiaotong University, Chongqing, China; 6 State Key Laboratory of Engineering Materials for Major Infrastructure, Sobute New Materials Co., Ltd., Nanjing, China; Henan Polytechnic University, CHINA

## Abstract

With the continuous increase in urban road usage, various cracks often appear on the road surface, which may pose a threat to traffic safety. Presently, road inspection is still primarily limited to manual methods, which suffer from low efficiency, limited accuracy, and subjective judgment. To enhance the efficiency of road crack detection, the paper designs an innovative detection technology that fuses U-net and ResNeXt networks. The results showed that the proposed method achieved superior detection performance on horizontal and vertical cracks. While its recognition and classification capabilities for other types of cracks and block cracks need improvement, it still demonstrated significant overall classification performance. Compared with numerous detection methods, the performance of the proposed method was notably superior. The peak memory efficiency of the video memory of this method is controlled within 2.1GB. This indicates that in practical applications, the proposed method can provide accurate information on road surface cracks, making it easier for workers to take corresponding remedial measures. In summary, the proposed urban road surface crack detection method can be integrated into intelligent transportation systems, providing technical support for real-time monitoring and predictive maintenance of road conditions.

## 1. Introduction

The rapid development of the Internet economy has made regional trade activities increasingly frequent, which has brought new challenges to the road traffic system [1]. Although China has made significant progress in road construction and maintenance, some roads with longer service lives still face various challenges in terms of road surface issues [2]. Road surface problems are usually caused by factors such as vehicle overweight, natural conditions, conventional wear and aging, and manifest as various visible damages to the road surface, such as horizontal, vertical, blocky, cracking, etc. [3]. Road cracks are early signals of road aging, and timely detection of cracks is crucial to prevent further deterioration [4]. Minor road damage may reduce the smoothness of vehicle travel and affect driving comfort. If timely maintenance of these minor issues is neglected, they may gradually deteriorate. Once road damage becomes severe, it may pose risks to the security of drivers and other road users. Therefore, regular maintenance and management of roads at all levels are important for ensuring road traffic safety and safeguarding public life and property. Through continuous maintenance and supervision, accidents can be prevented and the service life of roads can be improved. Meanwhile, through regular inspections and timely repairs, maintenance costs can be significantly reduced. Studies have shown that after implementing preventive maintenance, the average service life of road surfaces is extended by 5 years, while maintenance costs are saved by about 30% [5]. In the current practice of Road Surface Crack Detection (RSCD), the mainstream method still mainly relies on manual inspection, which has problems such as low efficiency and high accuracy affected by subjective factors. Despite the rapid development of AI-based automated detection technology, which demonstrates the potential for high precision and efficiency, its popularity and robustness in practical large-scale engineering applications still need to be further improved. Therefore, to enhance the accuracy of RSCD, the study proposes an innovative detection technique. This technology integrates U-net and ResNeXt networks, aiming to utilize advanced technology to timely and accurately detect and evaluate road damage.

More scholars are conducting research on the use of U-net. Wang H et al. developed a semantic segmentation framework that combines Channel Transformer (CTrans) and U-Net to address the challenges of U-Net in global multi-scale context modeling, UCTransNet. UCTransNet has achieved more accurate segmentation performance on different datasets, with significant improvements compared to traditional architectures [6]. Ghosh S et al. established an improved U-Net architecture for automatic evaluation and segmentation of brain MRI images. By analyzing the TCGA-LGG dataset in the TCI archive, it was found that this architecture outperformed common state-of-the-art CNN-based methods [7]. Meena S R proposed the potential of using U-Net and Machine Learning (ML) methods to automatically detect landslides in the Himalayas, addressing the issue of differences in mapping preferences caused by manual interpretation in event-based landslide inventories. The U-Net model trained with a patch size of 128 × 128 pixels produced the best MCC results on dataset-1 [8].

Many scholars have studied transformer-based models and self-supervised learning for crack detection. Huang et al. proposed an improved self-supervised learning technique for crack detection-self-attention intensive contrast learning. This method incorporated self-attention-related projection heads into the reinforcement contrastive learning architecture of self-supervised learning to obtain spatially continuous information of adjacent strata and utilized a mask region convolutional neural network for training. Experimental results showed that, based on the improved self-supervised learning framework, the average accuracy in crack detection reached 96.70%, 81.04%, and 94.67%, respectively, which was superior to the traditional method [9]. Lui et al. proposed a self-supervised learning model based on image features for crack detection. The model helped machine vision focus on the defect area in crack detection through an image fusion method based on defect-related feature extraction and used these features to generate pseudo-tags for self-supervised learning. Self-supervised learning combined the advantages of supervised and unsupervised learning to retain information about defects without sample labels. The actual case study results showed that the IFSSL model could effectively detect and locate cracks, improving the automation level of quality inspection [10]. Pandiyan et al. proposed a framework based on self-supervised learning for real-time quality monitoring of laser-directed energy deposition processes for crack detection. Combining convolutional neural network and converter architecture, an embedded vision system was used to monitor the characteristics of the melt pool. The process area images under different laser modes could achieve self-learning without the need for ground reality labels. By installing a coaxial charge-coupled device camera for image processing of the titanium powder deposition process, this framework achieved high classification accuracy and verified the effectiveness of self-supervised learning in crack detection and quality assessment [11].

A large number of scholars have developed crack detection technology based on artificial intelligence technology. Zhang J and Ding L developed a real-time detection framework based on artificial intelligence technology to address the problem that it was difficult to balance speed and accuracy in pavement crack detection on edge artificial intelligence mobile devices. This framework adopted a lightweight knowledge distillation network to improve the crack segmentation accuracy and perceived the hybrid distillation module through instance-aware integration of features and relationships. The results showed that the proposed method significantly improved the efficiency of automated crack detection and evaluation [12]. Zhang J et al. developed an automatic sealing robot based on artificial intelligence technology in response to the problems of crack segmentation refinement and insufficient sealing control accuracy in pavement crack detection in automatic sealing. The robot adopted a crack refining network to optimize the crack mask through the diffusion process. The results showed that the proposed method significantly improved the automation efficiency and robustness of crack detection and sealing [13]. The core of FFA adopts anisotropic diffusion filtering to enhance the crack features and extracts the crack regions through adaptive threshold segmentation. This method analyzes the directionality of local texture by constructing structural tensors and uses nonlinear diffusion equations to suppress noise while preserving the integrity of crack edges [14]. CrackForest is an automatic RSCD framework based on random structured forests, known for its high accuracy and speed [15]. The CrackForest method achieved an F1 value of 85.24% on the CFD dataset, indicating that it has a certain comprehensive detection capability. However, its recall rate is relatively low, at 83.7%, and its accuracy is 84.2%, suggesting a high risk of missed detections, which is a significant hazard in road safety inspections and cannot be ignored. Although the FFA method is theoretically beneficial for edge preservation based on anisotropic diffusion, its accuracy fluctuates greatly and is overall low in practical evaluations. The average accuracy is 78.6%, the recall rate is 68.4%, and the F1 value is 73.2%, resulting in a large number of false alarms, which will increase the burden of manual review in practical applications.

Overall, in recent years, the development of road crack detection technology based on artificial intelligence has been rapid, with various high-precision and high-efficiency methods emerging. For example, using self-supervised learning to reduce annotation dependency, designing lightweight models adapted to edge computing, building automated detection and repair closed-loop systems, exploring Transformer architecture to enhance global modeling capabilities, etc. Although U-net and ResNeXt networks have been widely used and performed well in image segmentation and classification, the research on deeply integrating the two to simultaneously achieve high-precision segmentation and fine-grained classification of urban road cracks is relatively limited. Existing methods often focus on single tasks (segmentation or classification) or specific optimization directions (such as speed or robustness in specific scenarios), lacking comprehensive solutions that integrate efficient segmentation, accurate classification, and easy deployment. This study proposes an urban RSCD method based on U-net and ResNeXt. The encoder-decoder structure and jumper connections of U-net can effectively integrate multi-scale features, particularly adept at identifying irregular targets such as cracks and achieving high-precision pixel-level localization. ResNeXt enhances feature diversity through grouped convolution, significantly improving the classification ability for crack morphology changes while maintaining computational efficiency. In the study, the convolutional units of U-net were replaced with ResNet residual blocks to construct the U-ResNet segmentation network. The residual structure was utilized to alleviate gradient vanishing and enhance feature extraction. The segmentation results are then input into the network containing the ResNeXt classification module for crack type identification, where ResNeXt improves the classification performance through grouped convolution. The contribution of the research lies in the automated identification and evaluation of road surface damage, reducing the need for manual inspections and thus lowering costs and time consumption. Secondly, accurate crack detection information helps to allocate maintenance resources reasonably, prioritize the treatment of severe cracks, and thus make more effective use of limited maintenance budgets. The innovation lies in the fusion of two advanced Deep Learning (DL) network architectures, U-net, and ResNeXt, which can more effectively extract features of road cracks and quickly process large amounts of data, significantly improving detection efficiency.

## 2. Methods and materials

### 2.1. Evaluation indicators for road cracks

For the fieldwork conducted in this study, the necessary permits were obtained from the relevant authorities. The full name of the authority that approved our access to the field site is “New-type Rail Transit Design and Research Institute of China Railway Siyuan Survey and Design Group Co., Ltd.”. The permit specifically allowed to carry out data collection and sample collection. The research strictly adhered to all the terms and conditions set out in the permit, including any relevant restrictions or guidelines such as specific time frames for access, environmental protection measures.

**Fig 1:**
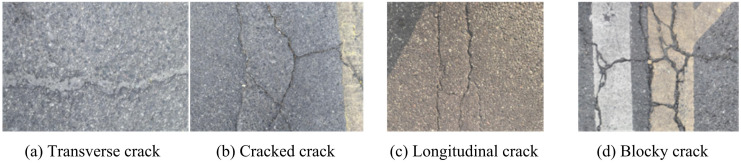
Common types of pavement cracks on urban asphalt roads (Source from: The author filmed it themselves; Chengdu, Jinxiu Avenue, longitude 103 ° 41 ′ −103 ° 55 ′ E, latitude 30 ° 36 ′ −30 ° 52 ′ N).

In the construction of urban roads, the pavement is mainly divided into asphalt pavement, cement concrete pavement, and other types based on the materials used [16]. With the continuous promotion of urban road construction in China, asphalt pavement has been widely adopted due to its structural advantages. There are two main types of cracks that appear on asphalt concrete pavement: load type cracks and non-load type cracks [17]. These two types of cracks may take on different forms, including linear cracks, horizontal cracks, grid-like cracks (similar to turtle shell cracks), and blocky cracks. The shape and distribution of these cracks can provide important information for evaluating road conditions. The common types of cracks on urban asphalt road surfaces are shown in Fig 1.

Fig 1 (a) −1 (d) correspond to transverse cracks, cracked cracks, longitudinal cracks, and blocky cracks. This study investigates and categorizes road surface cracks to comprehensively assess and determine the degree of damage to the road surface. The commonly used indicator for determining the degree of cracks is the Pavement Condition Index (PCI). PCI can reflect the damaged condition of asphalt pavement and is commonly used to characterize the integrity of the pavement. The specific calculation formula may involve road surface Damage Rate (DR) and some calibration coefficients, as shown in equation (1) [18].


$$\text{PCI}=100-\alpha_{\text{0}}{\text{DR}}^{\alpha_{\text{1}}}$$ (1)

In equation (1), $\alpha_{\text{0}}$ is a constant term. $\alpha_{\text{1}}$ is another coefficient used to adjust the degree of influence of DR values in the formula. From this, the calculation formula for DR can be inferred, as shown in equation (2).


$$\text{DR}=100\times \frac{\sum_{i=1}^{k}w_{i}A_{i}}{A}$$ (2)

In equation (2), $A_{i}$ is the size of the damaged area of Class i pavement cracks. A is the total area of the road surface region. $w_{i}$ is the coefficient of the relative importance or severity of Class i pavement damage. k is the gross of damage kinds with varying degrees of damage (light, medium, heavy). After mastering the method for determining the degree of pavement cracks in this study, it is needed to assess the performance of the calculation method in order to provide a solid data foundation for subsequent detection research. This study selects Precision, Recall, and F-measure as evaluation metrics for the detection algorithm. The accuracy calculation is given by equation (3) [19].


$$\text{Precision}=\frac{\text{TP}}{\text{TP}+\text{FP}}$$ (3)

In equation (3), TP is the true example, which is the positive sample correctly predicted by the model. FP is a false positive example, which means the model incorrectly predicts negative samples as positive samples. The calculation expression for Recall is given by equation (4).


$$\text{Recall}=\frac{\text{TP}}{\text{TP}+\text{FN}}$$ (4)

In equation (4), FN is a false counterexample, that is, the model incorrectly predicts a positive sample as a negative sample. F-measure is an indicator utilized to evaluate the performance of classification models, which combines precision and recall to provide a comprehensive evaluation criterion [20]. The calculation of F-measure is shown in equation (5).


$$\text{F-measure}=\frac{2\times \text{Precision}\times \text{Recall}}{\text{Precision}+\text{Recall}}$$ (5)

### 2.2. DL detection algorithm

**Fig 2:**
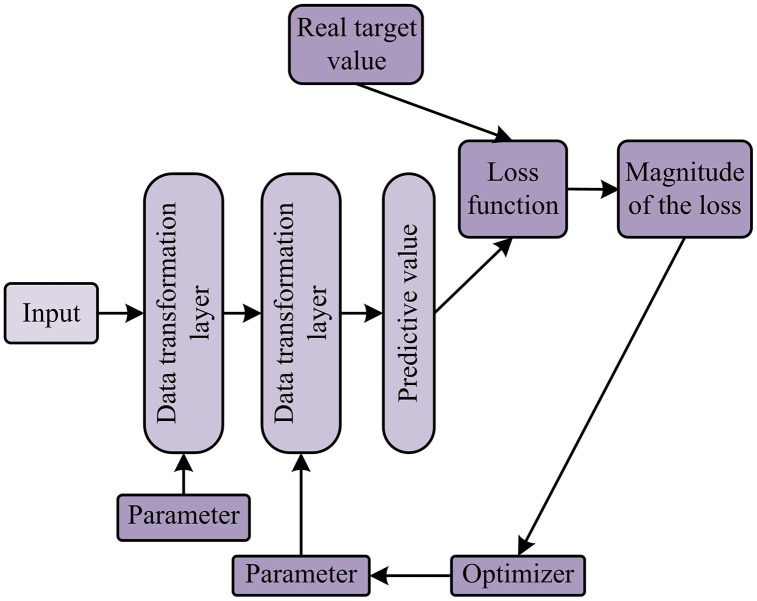
Flowchart of DL principle.

At present, the core algorithms of RSCD are all based on DL construction. It is a branch of ML that is based on the learning algorithms of Artificial Neural Networks (ANN), particularly those layers with multiple nonlinear transformations [21,22]. The concept of DL originated from the research of ANN, which attempts to simulate the neural network of the human brain to achieve advanced feature learning of data [23,24]. The learning principle of DL is shown in Fig 2.

In Fig 2, the principle of DL is first to randomly initialize the network parameters. Subsequently, through the forward propagation mechanism, the network generates predicted outputs.

### 2.3. U-NET crack image segmentation method

In RSCD, the combination of digital images and DL algorithms is highly valued due to its high degree of automation and fast detection speed. The key to achieving efficient crack detection lies in the accurate segmentation of crack images. For this purpose, a crack image segmentation method integrating U-net and ResNet is proposed in the study. ResNet is a deep network model, and its core structure is composed of multiple residual blocks stacked together. Each residual block contains two Convolutional Layers (ConvLs), followed by batch normalization processing and ReLU activation function operations in sequence [25]. The residual blocks are connected through quick connections. This design allows the gradient information to be directly transferred from the input layer to the output layer, effectively alleviating the common gradient vanishing problem in deep networks and ensuring the training stability of the network. On the other hand, U-net is a network architecture specifically designed for the image segmentation task. Its characteristic lies in the unique U-shaped structure, which consists of two parts: a symmetrical encoder and a decoder [26]. Road crack image segmentation is essentially a binary classification problem, that is, to determine whether each pixel in the image belongs to a crack. Therefore, the study adopts Binary Cross-Entropy as the loss function for model optimization, and its mathematical expression is shown in equation (6).


$$Loss=-\frac{\text{1}}{n}\sum_{i=1}^{n}\left[y_{i}log\left(P_{i})+(1-y_{i}\right)\text{log}\left(1-P_{i}\right)\right]$$ (6)

**Fig 3:**
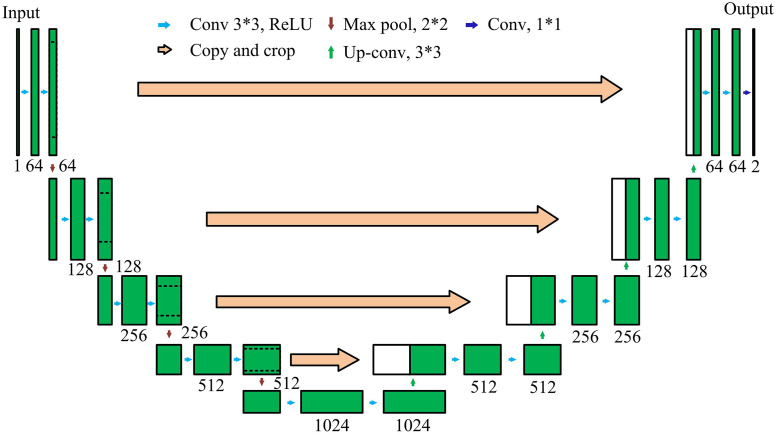
Schematic diagram of U-net structure.

In equation (6), n is the number of pixel values in the road surface image. $y_{i}$ is the label of sample i. $P_{i}$ is the probability of predicting the existence of cracks in sample i. In summary, ResNet mainly solves problems such as gradient dispersion between data layers, while U-net belongs to a network applied to image segmentation work. U-net has two parts: an encoder and a decoder, with a U shape. The framework of U-net is shown in Fig 3.

**Fig 4:**
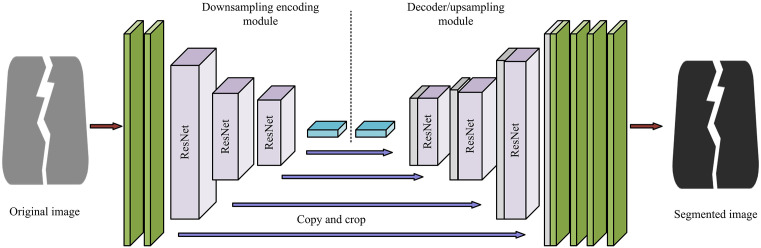
The operational structure of the U-ResNet.

In Fig 3, U-net achieves image segmentation through its symmetrical encoder-decoder structure and skip connections. In the decoding stage, the feature maps generated by each layer of the encoder are directly transmitted to the corresponding upsampling layer through skip connections. The design of the skip connection not only achieves feature fusion but also creates a shorter path for the backpropagation of gradients from the decoder to the shallow encoder. This mechanism effectively alleviates the common vanishing gradient problem in deep networks, ensures the stability of training, and enhances the network’s ability to learn fine-grained crack details. It is particularly suitable for tasks such as road crack segmentation. The operational structure of U-net is given in Fig 4.

In Fig 4, this study replaces the convolutional units of U-net with ResNet units, achieving the integration of high-level and low-level semantic information on a global scale. The U-Net architecture includes an encoder and a decoder, which implement downsampling and upsampling through a continuous residual structure. The encoder extracts multi-level features of the image through a continuous downsampling process, gradually transmitting information from the surface to the deep layers, and enhancing the ability to capture details such as road crack image texture through residual connections. This design also helps alleviate the common gradient vanishing problem in DL. The decoder is responsible for gradually upsampling the features and restoring them to the size of the original input image. At the same time, the decoder predicts the texture features of the crack image and optimizes it based on the difference between the predicted results and the actual cracks, ultimately outputting an accurate crack prediction image. Through this structure, U-net can integrate features of different scales and improve accuracy. The gradient calculation during the operation of U-net is shown in equation (7).


$$g_{i\;}=\nabla_{\theta }\;J\left(\theta_{i-1}\;\right)$$ (7)

In Equation (7), $\theta_{t}$ represents the random scalar parameter at the time step i, $g_{i\;}$ indicates the gradient of the objective function with noise over θ at the time step i, J(⬝) is the objective function, and ∇ represents the gradient operation. The calculation of the gradient exponential moving average at a certain moment during U-net operation is shown in equation (8).


$$m_{t}=\beta_{\text{1}}m_{t-1}+(1-\beta_{\text{1}}){\text{g}}_{t}$$ (8)

In equation (8), $m_{t}$ is the exponential moving average of the gradient, $\beta_{\text{1}}$ is the exponential decay rate, and ${\text{g}}_{t}$ is the gradient at time step t. The formula for calculating the exponential moving average of gradient squared is shown in equation (9).


$$\nu_{t}=\beta_{\text{1}}\nu_{t-1}+(1-\beta_{\text{1}})\overset{\text{2}}{\underset{t}{g}}$$ (9)

In equation (9), $\nu_{t}$ is the exponential moving average of the square of the gradient. Due to the initialization of $m_{t}$ being 0, the result will also tend towards 0. On this basis, this study performed bias correction on $m_{t}$ to reduce the impact of bias on training. The corrected $m_{t}$ calculation is shown in equation (10).


$$\hat{m_{t}}=m_{t}/(1-\overset{t}{\underset{\text{1}}{\beta }})$$ (10)

In equation (10), $\hat{m_{t}}$ is $m_{t}$ after deviation correction. Subsequently, this study also performs bias correction on $\nu_{t}$, and the calculation after correction is shown in equation (11).


$$\mathrm{\backslash stackrel}\wedge \nu_{t}=\nu_{t}/(1-\overset{t}{\underset{\text{2}}{\beta }})$$ (11)

In equation (11), $\mathrm{\backslash stackrel}\wedge \nu_{t}$ is $\nu_{t}$ after deviation correction. After combining $\hat{m_{t}}$ and $\mathrm{\backslash stackrel}\wedge \nu_{t}$ and updating the parameters, equation (12) can be obtained.


$$\theta_{t}=\theta_{t-1}-\alpha^{*}\hat{m_{t}}/(\sqrt{{\hat{\nu }}_{t}}+\varepsilon )$$ (12)

In equation (12), $\theta_{t}$ is the default learning rate with a value of 0.001. $\theta_{t}$ is a random scalar parameter for t time steps. The beginning of the next iteration is shown in equation (13).


$$\theta_{i}\;\leftarrow \theta_{i-1}\;-\alpha^{\text{2}}\frac{{\hat{m}}_{i}}{\sqrt{{\hat{v}}_{i}+\varepsilon }}\;$$ (13)

By using the above calculation formula, the goal of reducing the expected value of U-ResNet can be achieved, resulting in higher fault tolerance. To further improve the generalization ability of the proposed U-ResNet model, a comprehensive data enhancement strategy is introduced in the training phase. Considering the diversity of road crack morphology and potential environmental disturbances (such as shadows, stains, or uneven lighting), geometric transformations including random rotations, horizontal/vertical flips, and scaling are applied to simulate real-world changes in crack direction and size. In addition, luminosity distortions such as Gaussian noise injection, brightness adjustment (±20%), and contrast change (±15%) are introduced to improve robustness to illumination changes. These enhancements are dynamically applied during the training process to avoid overfitting and ensure that the model adapts to unknown scenarios.

### 2.4. Dual branch collaborative constraint classification network

**Fig 5:**
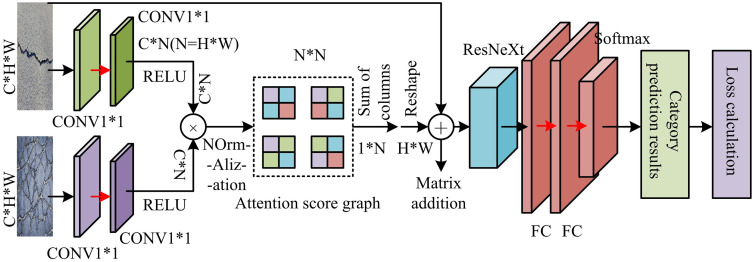
The operation structure of the DBCCN.

After the detection and segmentation of urban road surface crack images, they need to be classified and recognized. Crack classification is an important indicator for calculating pavement crack evaluation parameters, so this study further introduces a Dual Branch Collaborative Constraint Network (DBCCN). It is a DL model that processes different types of data through two different branches to improve the precision of feature classification and extraction [27]. Considering the complexity of crack morphology and the possible coexistence of static image and dynamic video data in actual detection scenarios, the dual-branch design of DBCCN can extract complementary features from spatial details (static images) and temporal variations (dynamic videos) respectively. The ConvL of branch one focuses on capturing the local texture and structural information of the cracks, while branch two analyzes the evolution characteristics of the cracks in consecutive frames through temporal modeling. The network structure is particularly suitable for processing multi-modal data, such as ultrasound images and contrast-enhanced ultrasound videos. These data can provide complementary information, thereby enhancing the diagnostic capability of the model. The operational structure of DBCCN is shown in Fig 5.

**Fig 6:**
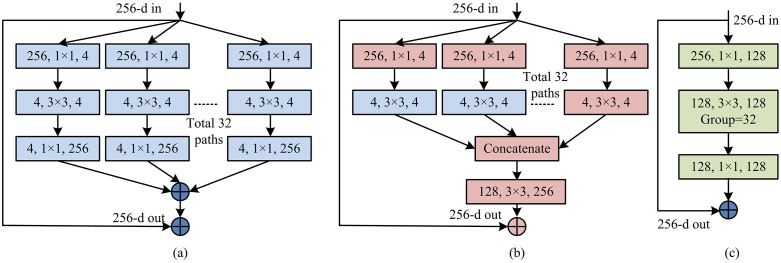
The operating structure of ResNeXt classification network.

In the DBCCN of Fig 5, one branch may focus on extracting features from static images, while the other branch focuses on extracting features from dynamic videos. This network extracts spatial features of static images and temporal features of dynamic videos through a dual-branch architecture. It introduces bilinear fusion and attention mechanisms to achieve cross-modal feature collaborative optimization, effectively enhancing crack information representation and suppressing background interference. The classification module is based on ResNeXt and utilizes its grouped convolution properties to efficiently process multi-source input features, ultimately outputting crack type recognition results. Compared with the traditional single-branch classification network, the DBCCN significantly enhances the discrimination ability of complex crack morphology through cross-modal feature collaborative optimization. However, traditional methods are limited in classification accuracy in dynamic video scenes due to the lack of a multi-source information fusion mechanism. The operational structure of the ResNeXt classification network is given in Fig 6.

In Fig 6, a module in ResNeXt performs a set of transformations, each on a low dimensional embedding, and its output is aggregated through summation. This network increases depth and width through repeated layers, and utilizes a split transform merge strategy to transform in an easily scalable manner. This design allows for expansion to any huge conversions without specialized design. The loss function calculation of ResNeXt network is shown in equation (14).


$$Loss=-\sum_{i=0}^{C-1}y_{i}log\left(p_{i}\right)=-log\left(p_{c}\right)$$ (14)

**Fig 7:**
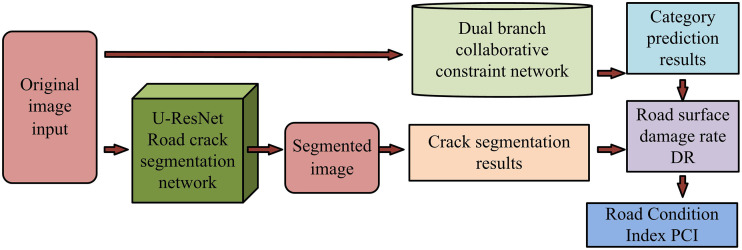
The urban road surface crack detection method.

In equation (14), C is the sample label and $p_{c}$ is the probability distribution of cracks. The complete process of the urban RSCD method researched and designed is shown in Fig 7. After the original pavement image is input, pixel-level crack segmentation is first achieved through U-ResNet, and the marked image is generated. Subsequently, DBCCN is innovatively adopted for crack type classification, and its parallel branch structure can handle multimodal features simultaneously. Finally, the severity of cracks is quantitatively evaluated based on DR and PCI indicators. A phased optimization strategy is adopted in the training stage. The classification network is fine-tuned by loading the ResNeXt-50 pre-trained weights (Adam optimizer, weight attenuation 1e-4). The segmentation network integrates cross-entropy and Dice loss (1:2) to solve the category imbalance and introduces the cosine annealing mechanism to dynamically adjust the learning rate (0.001 → 0.0001), significantly improving the robustness of the model.

Furthermore, to enhance the robustness of the model to complex disturbances in real scenes (such as water stains, oil stains, and their mixed stains after rain) more effectively, the study introduces the Generative adversarial Network (GAN) in the data augmentation stage. This GAN is trained to generate synthetic road images containing a variety of realistic and complex interference patterns [28]. Incorporating these synthesized images into the training set aims to enable the model to fully learn and distinguish crack features in various strong interference backgrounds during the training process, thereby significantly improving the model’s generalization ability in practical complex environments. Meanwhile, to alleviate sample imbalance (such as a small number of samples of massive cracks and cracked cracks) and enhance the extraction ability of key morphological features, the channel attention mechanism is introduced in the ResNeXt classification module in the study. This mechanism can adaptively enhance the characteristic response to the crack discriminative region. Furthermore, the Focal Loss is adopted to replace the standard cross-entropy loss function. By reducing the weights of easily classified samples (such as the background) and focusing on difficult-to-classify samples (such as fine or complex cracks), the training process of the classification network is optimized and the overall classification accuracy is improved.

## 3. Results

### 3.1. Image segmentation and detection analysis of pavement crack

In order to verify the more accurate recognition effect of the urban road surface crack detection method proposed by the research institute and ensure the repeatability of the experiment, all training and testing were conducted in a unified software and hardware environment. The hardware platform mainly uses workstations equipped with NVIDIA GeForce RTX 3090 GPU (24GB of video memory), and some edge deployment verification is completed on the NVIDIA Jetson AGX Xavier platform. The software environment is based on Ubuntu 20.04 LTS operating system, using Python 3.8 programming language and PyTorch 1.12.1 deep learning framework. The model training uses the Adam optimizer, with hyperparameters set as follows: the first-order and second-order momentum decay coefficients are 0.9 and 0.999, respectively, the numerical stability term is 10^−8^, the base learning rate is set to 0.001, and the cosine annealing scheduling strategy is applied to attenuate it to 0.0001 during the training period, with a weight decay coefficient of 0.0001. The batch size for both the segmentation network and the classification network is set to 4, and the training cycle is 80 rounds. All random processes use fixed random seeds, i.e., seed = 42, to ensure consistency of results.

To further ensure authenticity and reliability, the experiment introduces two datasets: Crack500 and Computational Fluid Dynamics (CFD) [29,30]. The Crack500 dataset is a specialized dataset for detecting and identifying concrete cracks, containing 500 images of concrete cracks. CFD contains 118 annotated crack images with a resolution of 480 × 320. Compared with other datasets, Crack500 covers the continuous scale distribution from slender micro-cracks to large through cracks. Its multi-scale characteristics can comprehensively verify the generalization ability of the model for crack morphology changes. The high-precision manual annotation and uniform resolution of CFD provide a reliable benchmark testing platform for the algorithm, avoiding evaluation deviations caused by annotation noise or size differences.

For the Crack500 dataset, it is randomly divided into training set, validation set, and testing set in proportions of 70%, 15%, and 15%. Due to the small sample size of the CFD dataset, in order to ensure the statistical reliability of the test set, 60% was used for training, 20% for validation, and 20% for testing. All data undergoes a standardized preprocessing process before being input into the model to ensure consistency and reproducibility of the experiment. The subsequent collection of the mixed dataset was carried out using an industrial camera fixed to the vehicle chassis, model: Daheng MER2–503-36U3M, with a resolution of 3840 × 2160, under natural lighting conditions. The camera lens was vertically downward about 1.2 meters from the ground, with a frame rate set to 30 fps, and all digital enhancement functions were turned off to maintain the original features of the image. The original images of public datasets (Crack500, CFD) and mixed datasets are first uniformly scaled to 1024 × 512 pixels, and bilinear interpolation is used to maintain the continuity of crack texture. Subsequently, the image is converted from RGB space to grayscale image to reduce channel dimension and enhance the contrast between cracks and road background. To further enhance the robustness of the model to changes in lighting and noise, histogram equalization was applied to the images during the training phase to enhance global contrast, and Gaussian noise with a standard deviation of 0.01 was randomly injected to simulate sensor noise in real environments. Finally, all pixel values are normalized to the [0,1] interval to accelerate the convergence of the model training process. This series of preprocessing operations aims to build an input data stream that is close to real road conditions and has uniform specifications, laying a reliable data foundation for subsequent crack segmentation and classification tasks. The experiment first calculates the types and weights of urban road surface damage in the dataset, as shown in Table 1.

**Table 1:** Types of urban road surface damage and weight calculation results in the dataset.

Type	Damage name	Degree of damage	Unit of measurement (m^2^)	Weight of manual investigation	Automated detection conversion factor
1	Cracked crack	Mild	Area	0.6	1.0
		Severe		1.0	
2	Blocky crack	Mild	Area	0.6	1.0
		Severe		0.8	
3	Longitudinal crack	Mild	Length × 0.2m	0.6	2.0
		Severe		1.0	
4	Transverse crack	Mild	Length × 0.2m	0.6	2.0
		Severe		1.0	

**Fig 8:**
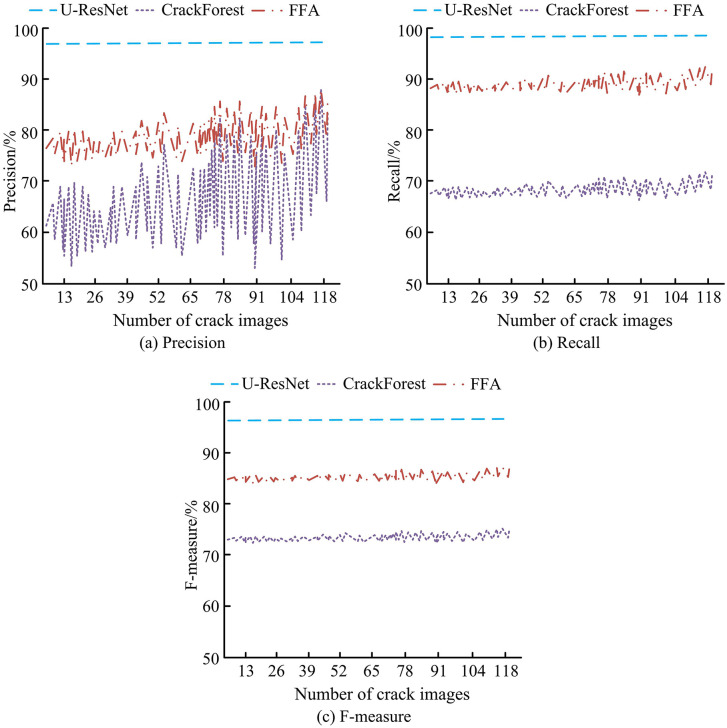
Performance comparison results of three methods.

In Table 1, when using automated detection technology, the area conversion factor for block repairs is 0.1. For strip repairs, the area conversion factor is 0.2. This conversion method helps to standardize the calculation of the area of road repair under different detection methods. The experiment evaluated the detection performance of U-ResNet on the CFD dataset. Additionally, two popular road detection methods were introduced as controls, namely FFA and CrackForest. The performance comparison of the 3 methods in given in Fig 8.

In Fig 8 (a), the precision of U-ResNet and CrackForest is relatively high and stable, while the precision of FFA shows significant fluctuations and is overall lower than that of U-ResNet and CrackForest. The precision of U-ResNet is as high as 97.81%, which is 19.25% higher than FFA. In Fig 8 (b), the recall rates of U-ResNet and CrackForest also show high stability, while the recall rate of FFA, although relatively stable, is overall lower than that of U-ResNet and CrackForest. The recall rate of U-ResNet is as high as 99.18%, which is 30.75% higher than FFA. In Fig 8 (c), the F-measure of U-ResNet remains at a high level, demonstrating its superior performance in crack detection tasks. Although CrackForest’s F-measure is slightly lower than U-ResNet, it still exhibits good performance. In contrast, FFA’s F-measure fluctuates significantly and its overall performance is not as good as U-ResNet and CrackForest. The F-measure value of U-ResNet is 98.52%, CrackForest is 85.24%, and FFA is 73.18%. In summary, U-ResNet outperforms CrackForest and FFA in crack detection performance on CFD datasets, particularly in precision and F-measure. This indicates that U-ResNet can timely detect and handle cracks, reduce maintenance costs, and improve resource utilization efficiency.

### 3.2. Analysis of experimental results on road crack image classification

**Fig 9:**
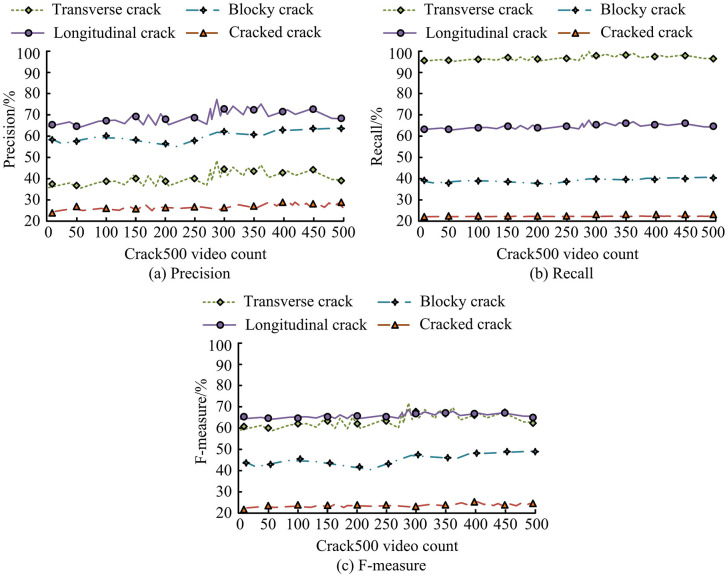
Classification performance of ResNeXt on four types of pavement cracks in the Crack500 dataset.

The experiment is conducted to verify the splitting effect of ResNeXt on detecting road crack images. The experiment sets the initial learning rate parameter to 0.001 and determines the beta1 parameter to be 0.900 and the beta2 parameter to be 0.988. The training cycle (Epoch) is 80 rounds, with each batch processing a data volume of 4. Secondly, this study selects Adam as the optimizer. Step 1 is to select the asphalt pavement crack images from Crack500, and then divide these images into equally sized grids of 3 × 3 pixels. The experiment then uses ResNeXt to classify four typical road surface cracks in Crack500, to verify the image classification performance of ResNeXt, as shown in Fig 9.

**Fig 10:**
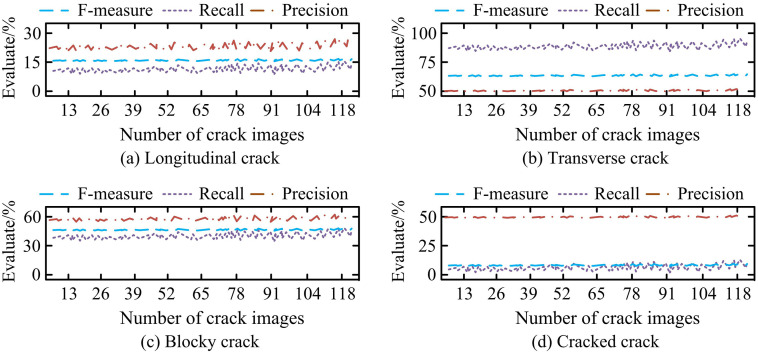
Classification performance of ResNeXt for 4 kinds of pavement cracks in CFD.

Fig 9 (a) shows the classification accuracy of ResNeXt for four types of cracks under different video counts. The precision of longitudinal and block cracks is relatively high, and it remains stable as the video count increases. The precision of transverse cracks and cracked cracks is low, but ResNeXt can still distinguish these types of cracks well. The highest classification precision is for longitudinal cracks, with a value of up to 66.25%. The above results may be due to the characteristics of the dataset itself and the complexity of the morphology of longitudinal cracks. The Crack500 dataset is derived from real road environments, where the background textures in the images are complex, and longitudinal cracks are often similar in height to linear textures, shadows, and even extensions of some horizontal cracks generated by road construction joints and asphalt materials, resulting in feature confusion and increasing classification difficulty. In addition, longitudinal cracks themselves have diverse shapes and may exhibit characteristics such as discontinuity, bending, or irregular expansion, further increasing the difficulty of the model learning their discriminative features. However, the research method still shows significant advantages in overall crack detection and classification tasks. The research method achieved an F1 score of 98.5% in the front-end segmentation stage, which means that the system has extremely high sensitivity for detecting longitudinal cracks and almost no missed detections. This is more critical than pure classification accuracy in practical engineering. Fig 9 (b) shows the recall performance of ResNeXt. The recall rate of transverse cracks is close to 100%, indicating that ResNeXt can identify almost all of these crack types. The classification recall rate of cracks is the lowest, with a value of 0.89%. Fig 9 (c) shows the overall performance of ResNeXt. The F-measure performance of longitudinal and transverse cracks is the best, indicating that ResNeXt has high accuracy and recall in detecting these types of cracks. The F-measure values of blocky and cracked cracks are low but still demonstrate the effectiveness of ResNeXt. The above data indicate that the ResNeXt network has the best classification performance on Crack500 for both horizontal and vertical cracks. It lacks recognition and classification performance for the other two types of cracks but still shows significant classification performance. This is crucial for road maintenance and safety. Through precise crack detection and classification, appropriate repair measures can be taken promptly to avoid potential traffic accidents and road damage. At the end of the experiment, ResNeXt is used on the CFD dataset to demonstrate its classification performance, as shown in Fig 10.

In Fig 10 (a), ResNeXt maintains a high level of F-measure, recall, and precision for longitudinal cracks, indicating that the network can effectively distinguish longitudinal cracks. In the transverse crack subgraph of Fig 10 (b), ResNeXt exhibits extremely high performance in detecting transverse cracks, with a recall evaluation index close to 100%. This means that the proposed network has extremely high precision and reliability in this type of crack detection. In the block fracture subgraph of Fig 10 (c), the evaluation index values of ResNeXt are slightly lower than those of transverse and longitudinal cracks, but overall it still maintains high performance. In the crack subgraph of Fig 10 (d), the classification performance of ResNeXt is poor, with a decrease in precision and recall, and the values of the three indicators do not exceed 50%. This might be because the cracks exhibit a high degree of complexity and irregularity in morphology, often presenting as fine, dense, and multi-directional network-like textures. Their visual characteristics can be highly confused with the background noise, stains, and even some block-like and network-like cracks of the road, thereby increasing the difficulty of classification decisions. Furthermore, it also objectively reflects the limitations of the current method in extremely challenging fine-grained classification tasks. To address the issue of relatively low classification accuracy, the research method can introduce an attention mechanism into the classification network, enabling the model to adaptively focus on the discriminative local features of the crack area and suppress the interference from complex backgrounds. At the same time, advanced loss functions such as Focal Loss will be adopted to alleviate the problem of class imbalance, allowing the model to pay more attention to the complex and difficult-to-classify crack samples. In summary, ResNeXt has high accuracy and stability in practical applications and can provide reliable data support for road maintenance, thereby improving road safety and service life.

### 3.3. RSCD simulation analysis of urban road surface damage identification and segmentation classification accuracy

**Fig 11:**
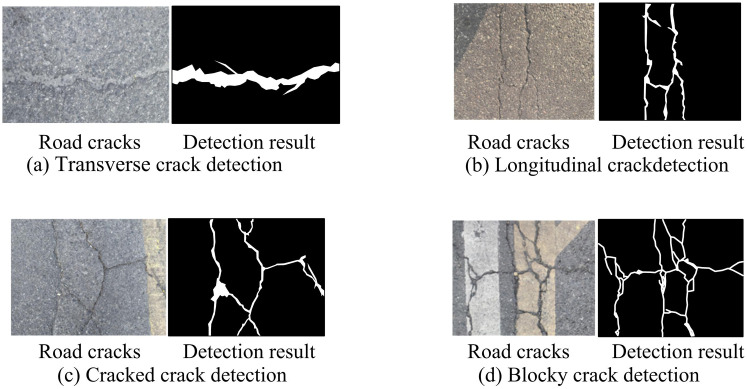
Segmentation and detection results of road surface crack images (Image source: The author filmed it themselves; Chengdu, Jinxiu Avenue, longitude 103 ° 41 ′ −103 ° 55 ′ E, latitude 30 ° 36 ′ −30 ° 52 ′ N).

To verify the engineering applicability of the proposed method, systematic simulation experiments are carried out in real road scenarios. The experiment selects the abandoned asphalt road in the suburbs of a certain city as the test site, and collects the road surface images through the vehicle-mounted industrial camera. The selected road is Jinxiu Avenue in Chengdu, located between longitude 103 ° 41 ′ −103 ° 55 ′ E and latitude 30 ° 36 ′ −30 ° 52 ′ N. This camera continuously collects for 6 minutes under typical daytime lighting conditions at 4K resolution and 30 fps, obtaining a total of 10,800 frames of video materials. In the sample construction stage, a rigorous three-stage screening process is adopted: Firstly, the pre-trained MobileNetV3 model is used to automatically identify frames with cracks. The introduced lightweight and pre trained MobileNetV3 classification model serves as the initial filter. It has undergone pre training on large general image datasets and possesses powerful general feature extraction capabilities. This step significantly reduces the workload of manual screening and ensures that the subsequent data samples used for fine annotation and model testing have clear research value. Secondly, filtering is carried out based on the quantization standards where the proportion of crack pixels exceeds 0.5% and the aspect ratio of the minimum peripheral rectangle is within the range of 1:10–10:1. Finally, the crack types are independently labeled by three road engineers, and only the samples with a labeling consistency of more than 90% are retained. Eventually, a test set containing 300 typical crack images is formed. This test set covers four types of damage: transverse cracks, longitudinal cracks, massive cracks, and cracking, with distribution ratios of 38%, 29%, 21%, and 12%, respectively. The simulation process consists of four core modules: During the image acquisition stage, a camera installed vertically 1.2 meters away from the road surface is used, combined with a 5500K color temperature LED auxiliary lighting system to ensure uniform illumination. In the real-time segmentation stage, the algorithm is deployed on the NVIDIA Jetson AGX Xavier edge computing platform. The inter-frame difference method is adopted to dynamically select key frames, and the input resolution of U-ResNet is adjusted to 1024 × 512 pixels. Combined with the sliding window strategy with a step size of 256 pixels, high-resolution images are processed. The classification evaluation stage is performed by the DBCCN network for multimodal feature analysis; The final calculation module generates quantitative evaluation based on the DR weight. To test the robustness of the model, Gaussian noise and raindrop occlusion combined interference are particularly injected into 20% of the samples. The standard deviation of the noise is set to 0.05, and the raindrop occlusion rate is controlled at 15%. The experiment extracts 300 road surface images with obvious cracks as analysis cases. The experiment conducted damage identification and image segmentation on crack types and parameters in actual scenarios, with an input size of 640 * 360 pixels, as shown in Fig 11.

**Fig 12:**
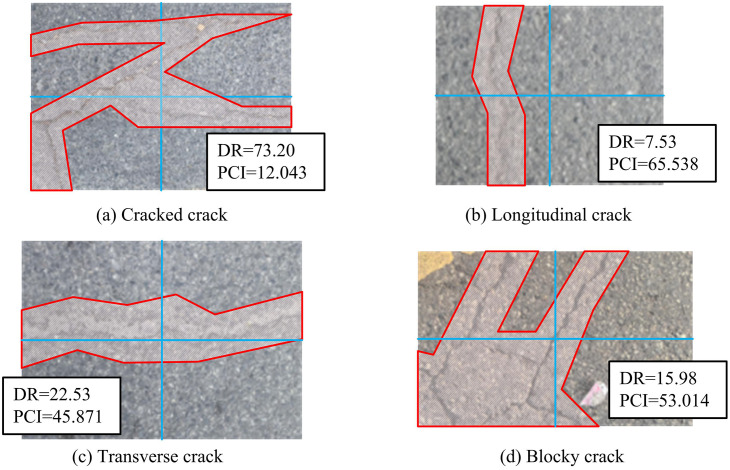
Classification results of ResNeXt classification network for road crack images (Image source: The author filmed it themselves; Chengdu, Jinxiu Avenue, longitude 103 ° 41 ′ −103 ° 55 ′ E, latitude 30 ° 36 ′ −30 ° 52 ′ N).

In Fig 11 (a), U-ResNet has higher detection accuracy for transverse cracks and can to some extent magnify the details of crack cracking. In practical applications, this is beneficial for operators to promptly detect cracks and take remedial measures to avoid further increase in crack stress. In Fig 11 (b), U-ResNet tends to extract the main crack contours for crack recognition and crack type segmentation. Compared with the data graph, the segmented crack image preserves the main crack patterns in the crack dense areas and reduces the interference of subtle cracks in the crack dense areas. In Fig 11 (c), U-ResNet achieves extremely accurate segmentation of cracks, without confusing road markings with crack edges, resulting in clear image segmentation and improved detection efficiency. In Fig 11 (d), for inconspicuous block cracks, the research method can accurately extract them and maintain consistency with the actual crack texture. In summary, the research method can accurately detect road cracks and segment images, which is consistent with the actual crack texture, indicating that the network has strong adaptability and robustness. Label DR, PCI, and damage types above each road crack image, and the classification results are shown in Fig 12.

**Fig 13:**
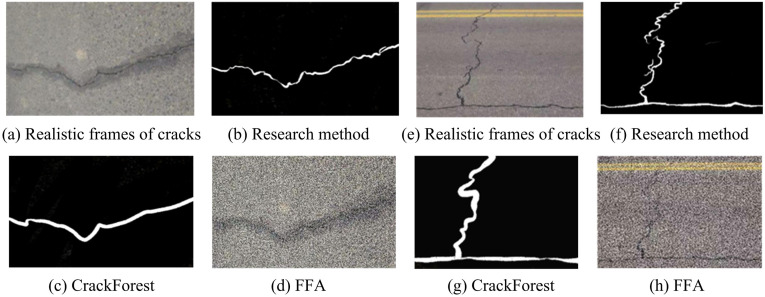
Simulation results of detection and classification using three methods (Source from: The author filmed it themselves; Chengdu, Jinxiu Avenue, longitude 103 ° 41 ′ −103 ° 55 ′ E, latitude 30 ° 36 ′ −30 ° 52 ′ N).

In Fig 12 (a), the DR evaluation value of the classified crack image is 73.20, and the PCI evaluation value is 12.043. The classified crack image will frame the actual crack range, which includes the actual crack area below the road surface. The degree of damage to road surfaces with cracks is relatively severe. The classification network is influenced by DR and PCI. The larger the DR value, the smaller the PCI value, making it easier to identify road surfaces with cracks. In Fig 12 (b), the DR evaluation value of the classified vertical crack image is 7.53, and the PCI evaluation value is 65.538. The degree of damage to road surfaces with vertical cracks is relatively mild. In Fig 12 (c), the DR evaluation value of the classified transverse crack image is 22.53, and the PCI evaluation value is 45.871. The degree of damage to transverse cracks on the road surface is relatively mild, but more severe compared to vertical cracks. In Fig 12 (d), the DR score of the classified block crack image is 15.98, and the PCI score is 53.014. The degree of damage to road surfaces with block cracks is relatively light, heavier than that of vertical cracks, and smaller than that of horizontal cracks. In summary, the proposed ResNeXt can determine crack types based on crack area, which is beneficial for image classification. In practical applications, this network can greatly improve detection and classification efficiency, and reduce labor costs. The experiment first performs crack detection and classification on 300 images, while comparing the CrackForest and FFA methods. The detection and classification results are shown in Fig 13.

Fig 13 shows the segmentation and classification results of road surface cracks in videos using three methods. Fig 13 (a) and (e) show transverse and longitudinal cracks. Fig 13 (b) and (f) show the segmentation and classification results of the research method. Consistent with the actual road crack frame image, the true crack distance and trend of the cracks have been restored, and interference elements have been eliminated. The detection of significant changes in image trends can provide accurate information on road cracks in practical applications, making it easier for workers to take corresponding remedial measures. Fig 13 (c) and (g) show the segmentation and classification results of CrackForest. It is consistent with the actual road crack frame image, but it does not restore the true crack distance and trend, but roughly summarizes the crack location and trend. Fig 13 (d) and (h) show the results of the FFA detection method. Compared with the actual road crack frame image, this result is relatively blurry, and only the crack position and trend can be vaguely seen. It cannot eliminate the interference elements in the picture, such as road indicator lines and stain marks. In practical application, it will greatly increase the workload of detection and classification, and cannot truly restore the road conditions, which may delay construction and remediation. To further comprehensively evaluate each detection method, 10 repeated experiments were conducted, and the statistical test results are shown in Table 2.

**Table 2:** Statistical test results.

Test method	IoU (%)	F1-score (%)	FNR (%)	FRR (%)
U-net and ResNeXt	92.4 ± 1.2	98.5 ± 0.3	0.82 ± 0.15	1.05 ± 0.28
CrackForest	78.1 ± 3.5	85.2 ± 1.8	14.8 ± 2.1	8.73 ± 1.92
U-net	76.3 ± 4.1	86.9 ± 1.5	13.1 ± 2.8	7.45 ± 2.15
VGG16 [17]	72.8 ± 5.0	84.3 ± 2.2	15.7 ± 3.3	10.21 ± 3.01
ResNeXt	70.5 ± 5.8	82.1 ± 2.7	17.9 ± 3.9	12.34 ± 3.87
FFA	68.2 ± 7.2	73.2 ± 4.5	26.8 ± 5.5	18.92 ± 4.62

Intersection over Union (IOU) is a core indicator for measuring the accuracy of image segmentation. It calculates the overlap area between the crack area predicted by the model and the true labeled crack area (Ground Truth), divided by the union area of these two areas [31,32]. The F1 score comprehensive evaluation of classification model performance is the harmonic mean of precision and recall [33]. The missed detection rate (FNR) refers to the proportion of actual positive samples that are incorrectly identified as negative; False positive rate (FRR) refers to the proportion of actual negative samples that are incorrectly identified as positive; The former measures the probability of false alarms, while the latter reflects the risk of missing targets. Table 2 shows that the research method performs the best in both comprehensive performance and safety key indicators, with an IoU of 92.4%, an F-value of 98.5%, an FNR of 0.82 and an FPR of 1.05%, both of which are the lowest, and the standard deviation is the smallest, corresponding to ± 0.15% and ± 0.28%, indicating that the detection results are highly stable and reliable. In contrast, the FFA missed detection rate is as high as 26.8% and fluctuates greatly, with a standard deviation of ± 5.5%, indicating significant safety hazards. Through in-depth analysis, it was found that the research model has a very low rate of missed detection for transverse cracks (<1%), but a high rate of missed detection for longitudinal cracks and fissures, reaching 12.3% ± 3.1% and 18.5% ± 4.7%, respectively. The main reason for the missed detection of these two types of niche cracks is low signal-to-noise ratio samples: one is extremely fine cracks with a width less than 3 pixels; The second is low visibility cracks with a contrast of less than 10; The third type is cracks partially covered by stains or markings. These samples result in insufficient feature extraction and fuzzy discrimination in the model. In the future, we can focus on enhancing the perception ability of models for fine-grained, low contrast, and occluded cracks, in order to prioritize the detection rate of high-risk cracks. To further confirm the effectiveness of each technical module of the research method, an ablation experiment is designed to compare the performance of models with different configurations on CFD datasets, as shown in Table 3.

**Table 3:** Ablation experiment.

Experimental configuration	Precision/%	Recall/%	F-measure/%
Basic model (U-Net only)	88.2	85.7	86.9
Basic model + data enhancement	92.1	91.3	91.7
Base model + ResNeXt	94.5	93.8	94.1
Base model + U-ResNet	90.2	87.5	88.8
Complete model (U-ResNeXt + Data enhancement)	97.8	99.2	98.5

Table 3 shows that in the absence of data augmentation and ResNeXt, the model is more sensitive to the interference of complex backgrounds, resulting in a higher mean square error (0.50) and an F-measurement value of only 86.9%. In the complete model, the joint optimization of U-ResNet and data augmentation achieves the optimal model performance, with the F-measurement value reaching as high as 98.5%. This indicates that the synergy of the two can significantly improve the robustness and accuracy of crack detection. The experimental results show that data enhancement and the introduction of ResNeXt optimize the model from the aspects of data diversity and feature representation, respectively, and their combined use can effectively deal with complex challenges in real road scenarios. The F-value difference between Base model+ResNeXt and Base model+U-ResNet is 5.3%, which directly reflects the key role of grouped convolution in crack detection tasks. This gap can be attributed to the multi-path grouped convolution structure of ResNeXt: it significantly enhances the network’s feature representation ability for complex crack shapes, such as irregular cracks and slender longitudinal cracks, through parallelization and diversified feature transformations, while the single residual path of standard ResNet has limitations in feature diversity. In terms of memory efficiency, the video memory usage of this method for single inference is only 1.8GB, which is more than 48.6% lower than traditional methods. Meanwhile, its peak memory is controlled within 2.1GB, which is 46.2% less than the traditional architecture (3.9GB).

To fully evaluate the generalization and robustness of the research method, a mixed large dataset containing 1,000 high-resolution road images is constructed, covering complex real-world scenarios. The data include lighting changes, weather disturbances, and road texture diversity. Among them, the lighting changes include strong light, low light, and back light (30% each). Weather disturbances include rainy, foggy, and snowy days (20% each). The diversity of road texture includes asphalt, cement, patched pavement, sand, and gravel pavement (25% each). The image collection area is from longitude 103 ° 41 ′ to 103 ° 55 ′ E and latitude 30 ° 36 ′ to 30 ° 52 ′ N. The research method is compared with the latest Modern segmentation technique and Detection model technology, as shown in Table 4. In comparative experiments, Modern segmentation technology is a mainstream fine-grained segmentation technique based on Transformer. This technology captures long-term dependencies through a global attention mechanism and is adept at handling complex textures, but has a high computational cost. Detection model technology is a hybrid architecture combining target detection and semantic segmentation. It locates the crack region through the region suggestion network and performs pixel-level segmentation, taking into account detection efficiency and accuracy. To systematically evaluate the deployment feasibility of this research method in resource-constrained environments, the study deployed it along with two advanced benchmark models on desktop GPUs (NVIDIA RTX 3090), vehicle-mounted edge computing platforms (NVIDIA Jetson AGX Xavier), and unmanned aerial vehicle inspection platforms (NVIDIA Jetson TX2) for testing. From this, we can obtain the overall performance comparison of different advanced methods on various deployment platforms, as shown in Table 4.

**Table 4:** Comparison of the overall performance of different advanced methods across various deployment platforms.

Method	Platform	Precision/%	Recall/%	F-measure/%	Inference speed/s	Frame processing time/ms	Peak Memory/FPS/WGB	Typical power consumption/FPS/WW	Energy efficiency ratio/FPS/W
Modern segmentation technique	Desktop GPU (RTX 3090)	83.2	85.5	84.3	29.7	33.7	3.9	350	0.085
	Edge device (Jetson AGX X)	78.5	80.1	79.3	8.2	122.0	2.8	30	0.273
Detection model technology	Desktop GPU (RTX 3090)	91.5	90.2	90.8	35.4	28.2	4.5	350	0.101
	Edge device (Jetson AGX X)	85.3	83.7	74.6	6.2	153.8	3.2	30	0.217
Research method	Desktop GPU (RTX 3090)	96.8	97.5	97.1	28.6	34.9	1.8	350	0.082
	Vehicle-mounted platform (Jetson AGX X)	95.1	96.3	95.7	9.3	107.5	1.8	50	0.310
	Unmanned aerial vehicle platform (Jetson TX2)	93.8	94.6	94.1	5.2	196.1	1.5	15	0.340

In Table 4, on the desktop GPU platform, all three methods can meet the basic requirements for real-time processing (frame rate higher than 24 FPS). Among them, the research method in this study achieved the highest overall detection accuracy (F1 value 97.1%) and the best memory usage efficiency (peak occupancy only 1.8 GB) while maintaining a high inference speed (28.6 FPS). Its single-frame processing delay (34.9 ms) is comparable to the detection model technology and significantly better than the computationally intensive modern segmentation techniques (58.1 ms), demonstrating the balance between accuracy and efficiency in an environment with abundant computing power. In resource-constrained edge deployment scenarios, the lightweight design adopted by this research method becomes even more prominent. On the vehicle platform (Jetson AGX Xavier), it can still maintain a real-time processing capability of 9.3 FPS and successfully control the peak memory occupancy at 1.8 GB, which is the same as the desktop end and much lower than the two comparison methods. More importantly, its energy efficiency ratio (0.310 FPS/W) is the highest among the comparison models, indicating that under the same power consumption constraints, the proposed method can complete more image processing frames, which is crucial for mobile inspection tasks powered by vehicle batteries. On the unmanned aerial vehicle platform (Jetson TX2), this method still maintains a usable 5.1 FPS processing speed and extremely low 1.5 GB memory occupancy, achieving the highest energy efficiency ratio (0.340 FPS/W) among all configurations. These data fully validate that by integrating the lightweight encoding and decoding structure of U-Net with the grouped convolution design of ResNeXt, this research method can simultaneously guarantee high-precision crack detection capabilities, satisfactory real-time performance, and excellent memory and energy utilization efficiency within strict computing and power consumption budgets, thus proving its practical engineering potential for integration into intelligent vehicle systems and unmanned aerial vehicle automatic inspection platforms.

## 4. Discussion

In response to the above problems, a network architecture integrating U-net and ResNeXt was studied and designed, which demonstrated significant advantages in the task of urban road crack detection.

In terms of segmentation and detection, U-ResNet achieved an F-measure of 98.52% on the CFD dataset. This is attributed to the following design: Firstly, the jump connection structure of U-Net effectively integrates multi-scale features and stabilizes gradient propagation, enabling the network to accurately capture the slender and irregular shapes of cracks. Second, the introduced residual structure alleviates vanishing gradients and ensures the training stability of deep networks. Compared with traditional methods such as CrackForest and FFA, U-ResNet has achieved a qualitative leap in accuracy and robustness, demonstrating the overwhelming advantage of deep learning in crack feature extraction.

In terms of classification performance, the ResNeXt classification network shows significant differences in the recognition accuracy of different crack types. Its recall rate for transverse cracks is close to 100%, but the classification accuracy for longitudinal cracks in the Crack500 dataset is only 66.25%, and the recognition effect for cracking is also not ideal. This reveals the inherent difficulty of the fine-grained crack classification task: longitudinal cracks are easily confused with road textures and linear stains; The morphology of cracking is complex and variable, and the number of samples may be relatively insufficient. This also demonstrates that in complex real-world scenarios, achieving high-precision crack semantic classification is more challenging than realizing crack detection with a high recall rate. Nevertheless, as an auxiliary decision-making link after detection, the output of the classification module, combined with the DR And PCI_sub-crack information provided by the segmentation module, can already provide data support far exceeding that of manual inspection for the ranking of maintenance priorities.

Simulation showed that the segmentation and classification results of the research method were consistent with the actual road crack frame images, restoring the true crack distance and trend of the cracks, and eliminating interference elements. The trend of the detected image changes significantly. In practical applications, research methods can provide accurate information on road surface cracks, making it easier for workers to take corresponding remedial measures. Although the proposed method has demonstrated high efficiency and accuracy in urban road crack detection, it still has the following limitations: (1) The scale and diversity of experimental data are relatively general, and it is difficult to cover real scenes such as extreme weather, complex lighting, and diversified pavement textures, which may affect the generalization ability of the model; (2) The model has not yet verified its computational efficiency and energy consumption in resource-constrained devices such as vehicles or drones; (3) The ResNeXt classification network has low accuracy in identifying massive cracks and composite cracks, which may result in missing detection due to unbalanced samples or insufficient feature extraction; (4) Existing data enhancement strategies do not fully simulate complex disturbances such as water stains and oil stains, which may reduce robustness in actual deployment. Future work can be promoted from four aspects. The first is to build large-scale data sets covering multiple environments and multi-modes (such as thermal imaging and liDAR) to improve model adaptability. The second is to reduce hardware dependence by adapting lightweight architecture optimization (such as network pruning and quantization) to the edge computing framework. The third is to introduce attention mechanism or improve loss function (such as focus loss) to alleviate classification imbalance. The fourth is to combine the generation of countermeasure networks, integrate crack data under complex interference, and enhance the anti-interference ability of the model. In addition, developing interpretability tools, such as significance maps, and working with municipalities to conduct field tests will move the technology from the lab to the engineering ground, providing more reliable support for smart transportation.

## 5. Conclusion

In response to the demand for automatic detection of cracks in urban roads, this research proposes a detection framework that deeply integrates U-net and ResNeXt networks. The main conclusions can be drawn as follows:

(1) In terms of segmentation: The U-ResNet network achieved an F1 score as high as 98.5% on public datasets, significantly outperforming traditional image processing and machine learning methods, demonstrating its high precision and robustness in pixel-level crack location.
(2) In terms of classification: The ResNeXt classification network demonstrates nearly perfect recognition ability for transverse cracks, but the classification accuracy for longitudinal and crease cracks needs to be improved.
(3) System Performance: The entire framework achieves a real-time processing speed of 28.6 FPS on complex mixed datasets, striking an outstanding balance between accuracy and efficiency, and has the potential for engineering deployment.

In summary, the research contribution lies in deeply coordinating the fine segmentation ability of U-net and the powerful classification ability of ResNeXt through the dual-branch structure of DBCCN, and constructing an end-to-end unified framework for crack detection and evaluation. Moreover, the proposed method can be integrated into the intelligent inspection system, providing a practical and feasible technical solution for the automatic and real-time detection and preventive maintenance of road diseases, which is conducive to reducing maintenance costs and improving road safety.

## Supporting information

S1 File Minimal Data Set Definition. (DOCX)
