# Supplementary material for: Urban road surface crack detection based on U-net and ResNeXt network
Source: PLoS One. 2026 Apr 21;21(4):e0347145. doi: 10.1371/journal.pone.0347145 (PMC13098964; doi:10.1371/journal.pone.0347145)
Supplement: S1 File — (DOCX) [file pone.0347145.s001.docx]

Minimal Data Set Definition

**The data in Figure 8**

| Evaluation index | Number of crack images | U-ResNet | CrackForest | FFA |
| --- | --- | --- | --- | --- |
| Precision | 13 | 97.81 | 55.04 | 73.53 |
|  | 26 | 97.82 | 64.72 | 76.84 |
|  | 39 | 97.83 | 57.63 | 74.06 |
|  | 52 | 97.82 | 72.31 | 82.72 |
|  | 65 | 97.83 | 66.42 | 78.43 |
|  | 78 | 97.83 | 53.41 | 83.15 |
|  | 91 | 97.84 | 65.64 | 72.62 |
|  | 104 | 97.84 | 63.82 | 74.17 |
|  | 118 | 97.83 | 75.64 | 80.13 |
| Recall | 13 | 99.18 | 67.82 | 86.42 |
|  | 26 | 99.19 | 68.36 | 87.05 |
|  | 39 | 99.20 | 67.61 | 86.95 |
|  | 52 | 99.19 | 69.63 | 88.45 |
|  | 65 | 99.20 | 68.74 | 89.23 |
|  | 78 | 99.21 | 65.24 | 85.04 |
|  | 91 | 99.21 | 65.07 | 84.65 |
|  | 104 | 99.22 | 68.06 | 87.62 |
|  | 118 | 99.22 | 68.73 | 90.14 |
| F-measure | 13 | 98.49 | 60.77 | 79.46 |
|  | 26 | 98.50 | 66.49 | 81.63 |
|  | 39 | 98.51 | 62.22 | 79.99 |
|  | 52 | 98.50 | 70.94 | 85.49 |
|  | 65 | 98.51 | 67.56 | 83.48 |
|  | 78 | 98.52 | 58.74 | 84.08 |
|  | 91 | 98.52 | 65.35 | 78.17 |
|  | 104 | 98.53 | 65.87 | 80.34 |
|  | 118 | 98.52 | 72.02 | 84.84 |

**The data in Figure 9**

| Evaluation index | Crack500 video count | Transverse crack | Blocky crack | Longitudinal crack | Cracked crack |
| --- | --- | --- | --- | --- | --- |
| Precision | 0 | 36.78 | 59.86 | 66.53 | 24.64 |
|  | 50 | 35.67 | 59.06 | 66.51 | 26.83 |
|  | 100 | 38.94 | 60.03 | 67.43 | 25.74 |
|  | 150 | 40.15 | 59.14 | 69.84 | 25.81 |
|  | 200 | 38.61 | 57.62 | 68.79 | 25.94 |
|  | 250 | 39.04 | 58.97 | 68.84 | 25.87 |
|  | 300 | 43.62 | 61.62 | 70.05 | 27.62 |
|  | 350 | 42.64 | 59.67 | 69.97 | 28.61 |
|  | 400 | 38.61 | 60.13 | 69.86 | 29.78 |
|  | 450 | 41.73 | 60.15 | 70.01 | 28.45 |
|  | 500 | 36.84 | 60.18 | 66.54 | 27.98 |
| Recall | 0 | 96.84 | 40.02 | 62.35 | 21.26 |
|  | 50 | 96.75 | 39.81 | 62.74 | 22.32 |
|  | 100 | 96.86 | 39.94 | 63.25 | 21.98 |
|  | 150 | 97.05 | 39.76 | 63.27 | 21.86 |
|  | 200 | 96.91 | 38.95 | 63.48 | 21.76 |
|  | 250 | 97.05 | 39.05 | 64.53 | 22.05 |
|  | 300 | 98.13 | 40.16 | 65.42 | 23.43 |
|  | 350 | 97.98 | 40.08 | 66.17 | 24.27 |
|  | 400 | 98.02 | 41.06 | 66.08 | 24.16 |
|  | 450 | 97.99 | 41.17 | 66.13 | 24.08 |
|  | 500 | 97.84 | 41.26 | 65.97 | 23.95 |
| F-measure | 0 | 53.31 | 47.97 | 64.37 | 22.83 |
|  | 50 | 52.12 | 47.56 | 64.57 | 24.37 |
|  | 100 | 55.55 | 47.97 | 65.27 | 23.71 |
|  | 150 | 56.80 | 47.55 | 66.39 | 23.67 |
|  | 200 | 55.22 | 46.48 | 66.03 | 23.67 |
|  | 250 | 55.68 | 46.99 | 66.62 | 23.81 |
|  | 300 | 60.39 | 48.63 | 67.66 | 25.35 |
|  | 350 | 59.42 | 47.95 | 68.02 | 26.26 |
|  | 400 | 55.40 | 48.80 | 67.92 | 26.68 |
|  | 450 | 58.53 | 48.88 | 68.01 | 26.08 |
|  | 500 | 53.53 | 48.96 | 66.25 | 25.81 |

**The data in Figure 10**

| Evaluation category | Number of crack images | Precision | Recall | F-measure |
| --- | --- | --- | --- | --- |
| Longitudinal crack | 13 | 22.43 | 12.35 | 15.93 |
|  | 26 | 22.38 | 12.16 | 15.76 |
|  | 39 | 22.36 | 13.07 | 16.50 |
|  | 52 | 24.16 | 12.09 | 16.12 |
|  | 65 | 23.87 | 12.27 | 16.21 |
|  | 78 | 23.95 | 13.24 | 17.05 |
|  | 91 | 21.06 | 11.97 | 15.26 |
|  | 104 | 22.13 | 13.14 | 16.49 |
|  | 118 | 27.64 | 13.85 | 18.45 |
| Transverse crack | 13 | 50.07 | 88.76 | 64.02 |
|  | 26 | 50.15 | 87.24 | 63.69 |
|  | 39 | 50.24 | 89.15 | 64.26 |
|  | 52 | 51.03 | 89.04 | 64.88 |
|  | 65 | 51.26 | 89.53 | 65.19 |
|  | 78 | 51.57 | 92.64 | 66.26 |
|  | 91 | 50.76 | 94.26 | 65.99 |
|  | 104 | 50.53 | 95.85 | 66.17 |
|  | 118 | 50.48 | 96.45 | 66.27 |
| Blocky crack | 13 | 58.75 | 38.65 | 46.63 |
|  | 26 | 58.69 | 37.16 | 45.51 |
|  | 39 | 58.94 | 40.23 | 47.82 |
|  | 52 | 60.04 | 38.61 | 47.00 |
|  | 65 | 59.81 | 37.94 | 46.43 |
|  | 78 | 57.62 | 43.65 | 49.67 |
|  | 91 | 60.12 | 42.17 | 49.57 |
|  | 104 | 60.08 | 43.13 | 50.21 |
|  | 118 | 60.26 | 45.36 | 51.76 |
| Cracked crack | 13 | 49.98 | 6.51 | 11.52 |
|  | 26 | 50.03 | 6.13 | 10.92 |
|  | 39 | 50.05 | 7.65 | 13.27 |
|  | 52 | 50.07 | 6.08 | 10.84 |
|  | 65 | 50.13 | 5.97 | 10.67 |
|  | 78 | 50.26 | 8.91 | 15.14 |
|  | 91 | 50.19 | 7.95 | 13.73 |
|  | 104 | 50.21 | 7.64 | 13.26 |
|  | 118 | 50.23 | 6.97 | 12.24 |
